# Supplementary material for: A model to identify urban traffic congestion hotspots in complex networks
Source: R Soc Open Sci. 2016 Oct 12;3(10):160098. doi: 10.1098/rsos.160098 (PMC5098960; doi:10.1098/rsos.160098)
Supplement: Supplementary material. Additional figures and validation of the model. [file rsos160098supp1.pdf]

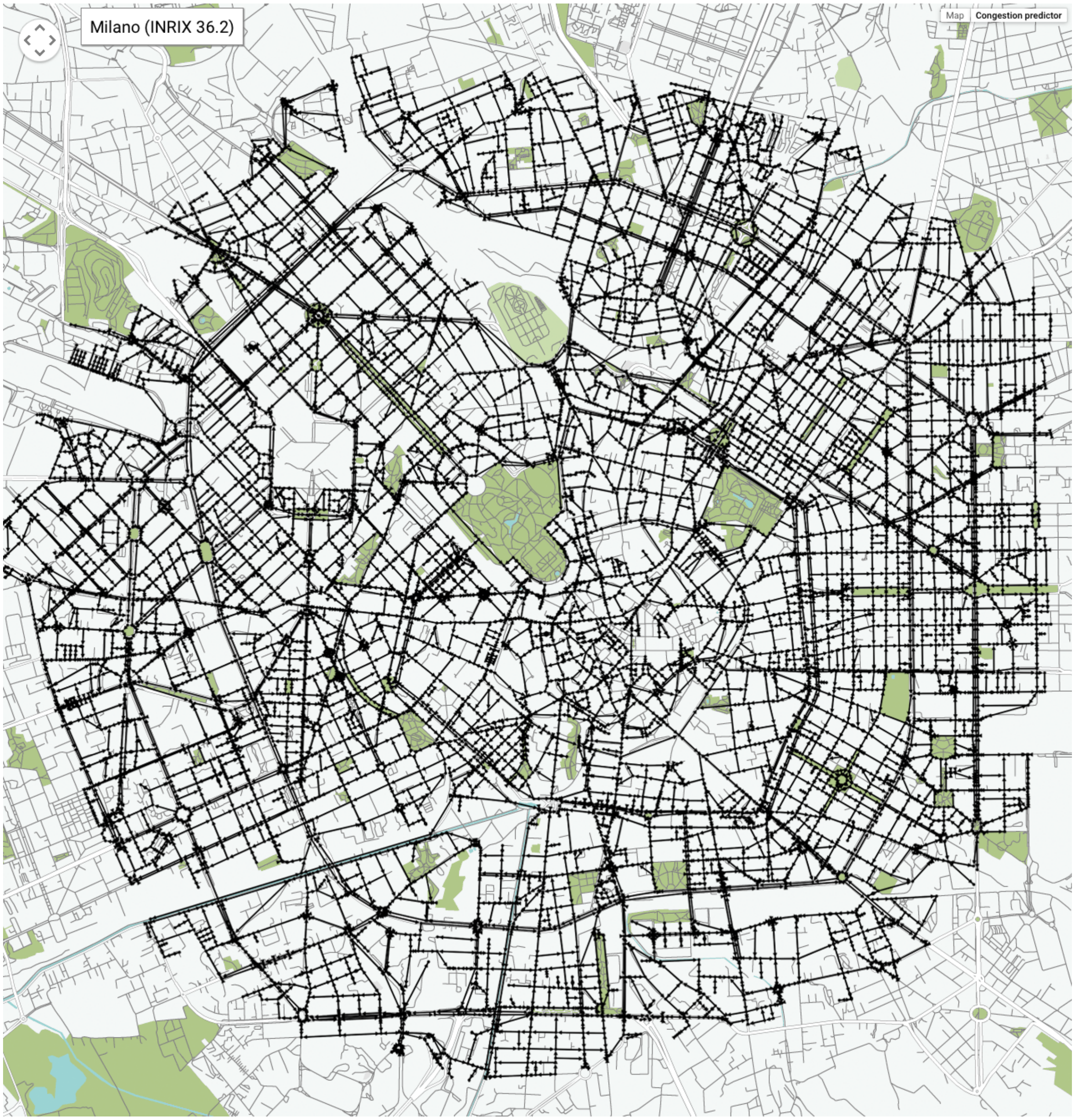

Supplementary Figure 1: Topology of the road network of Milan. Data gathered from Open Street Map (http://www.openstreetmap.org).

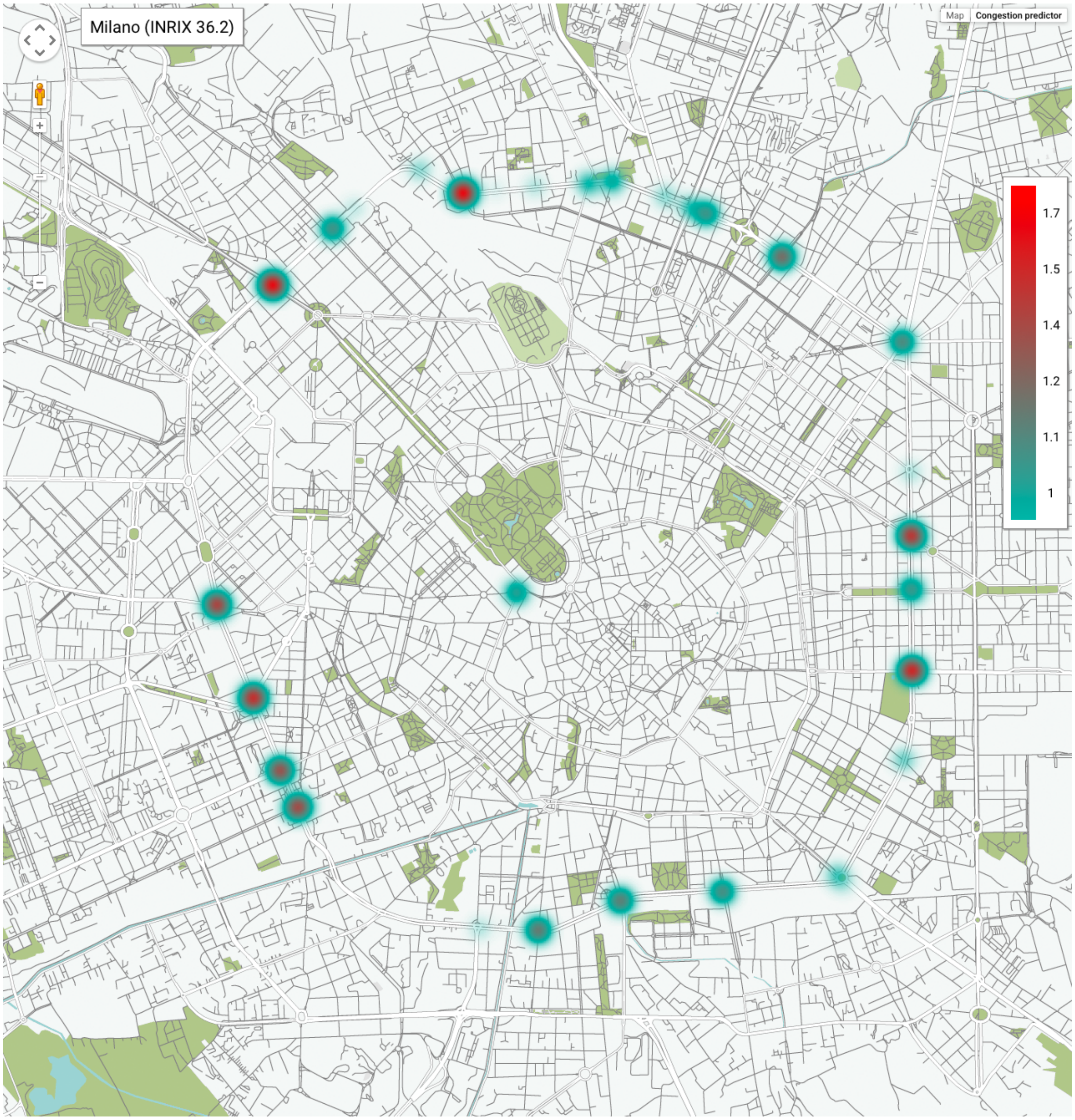

Supplementary Figure 2: Map of Milan showing the expected ratio between incoming and outgoing vehicles of each congested junction, $(\sigma_i + g_i)/d_i$. Several congested junctions might appear merged in the heat map. Model parameters have been obtained assuming homogeneously distributed source and destination locations and the required road traffic to obtain an order parameter η [1, 2] equivalent to the city INRIX value.

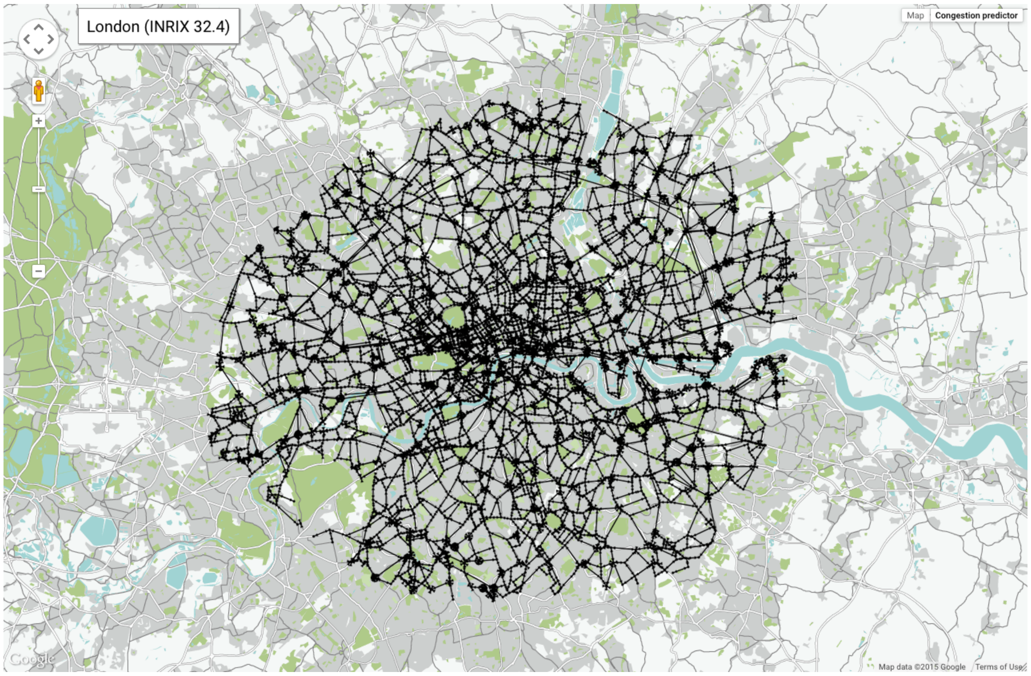

Supplementary Figure 3: Topology of the road network of London. Data gathered from Open Street Map (http://www.openstreetmap.org).

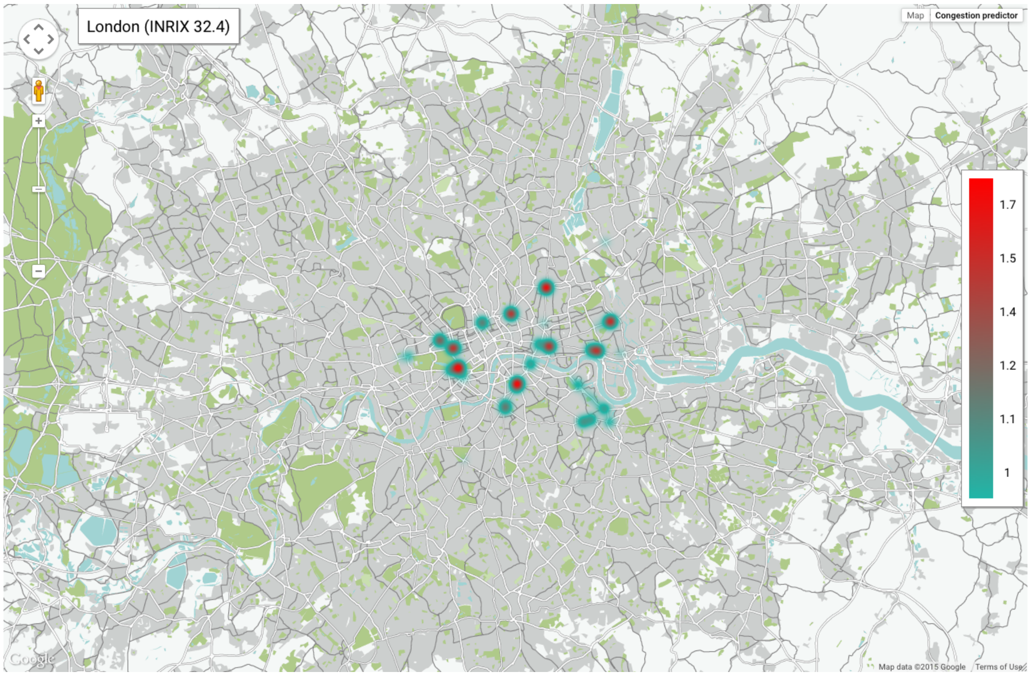

Supplementary Figure 4: Map of London showing the expected ratio between incoming and outgoing vehicles of each congested junction, $(\sigma_i + g_i)/d_i$. Several congested junctions might appear merged in the heat map. Model parameters have been obtained assuming homogeneously distributed source and destination locations and the required road traffic to obtain an order parameter η [1, 2] equivalent to the city INRIX value.

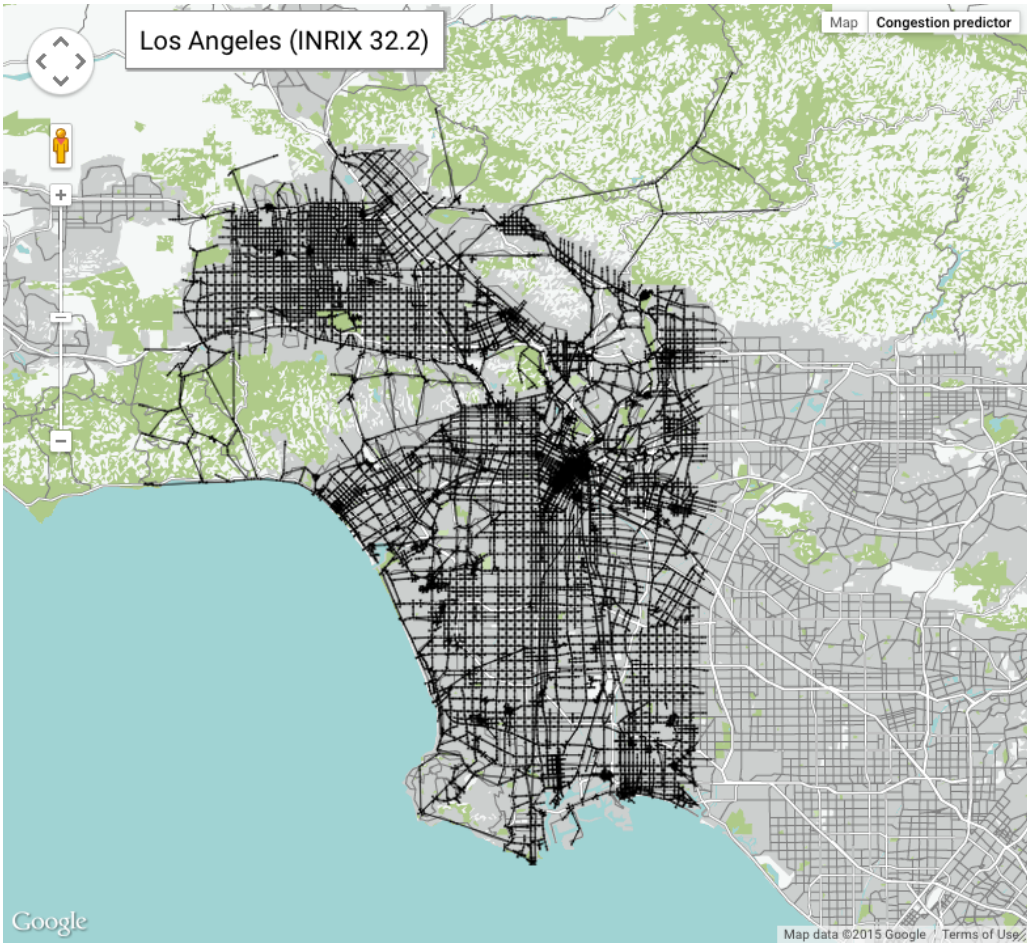

Supplementary Figure 5: Topology of the road network of Los Angeles. Data gathered from Open Street Map (http://www.openstreetmap.org).

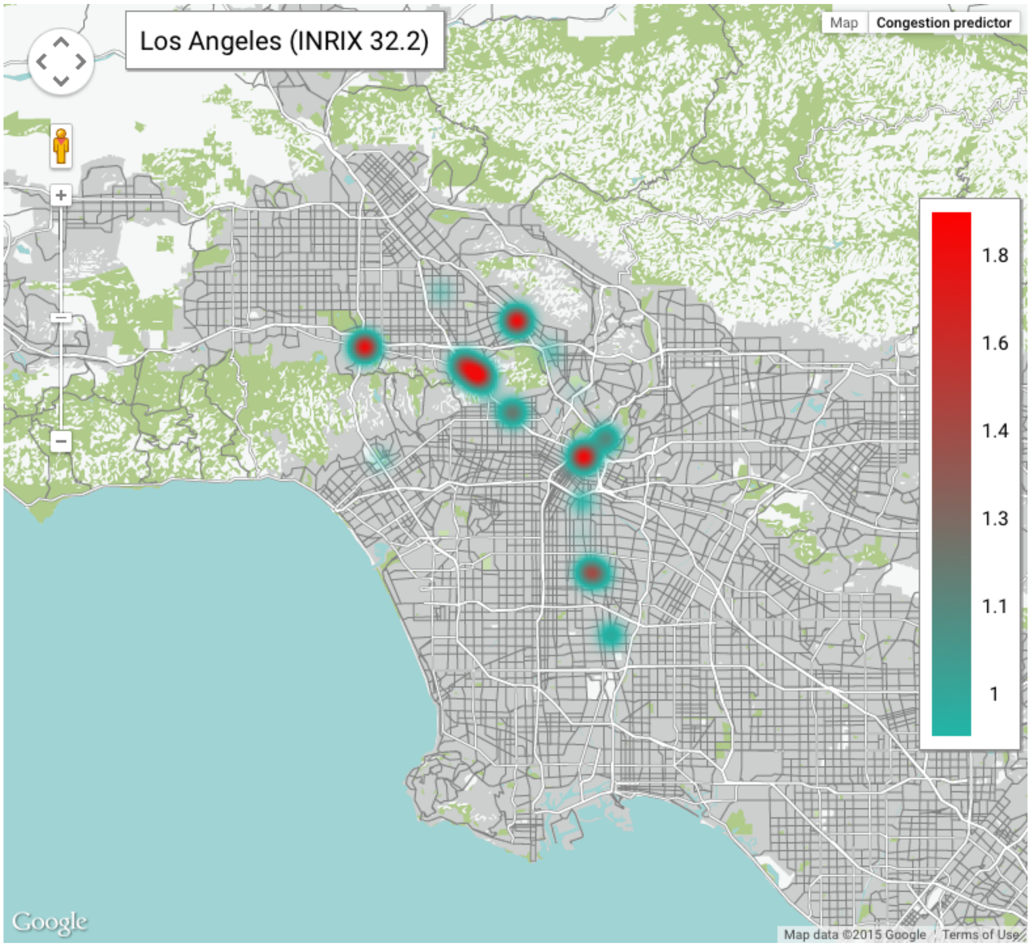

Supplementary Figure 6: Map of Los Angeles showing the expected ratio between incoming and outgoing vehicles of each congested junction, $(\sigma_i + g_i)/d_i$. Several congested junctions might appear merged in the heat map. Model parameters have been obtained assuming homogeneously distributed source and destination locations and the required road traffic to obtain an order parameter η [1, 2] equivalent to the city INRIX value.

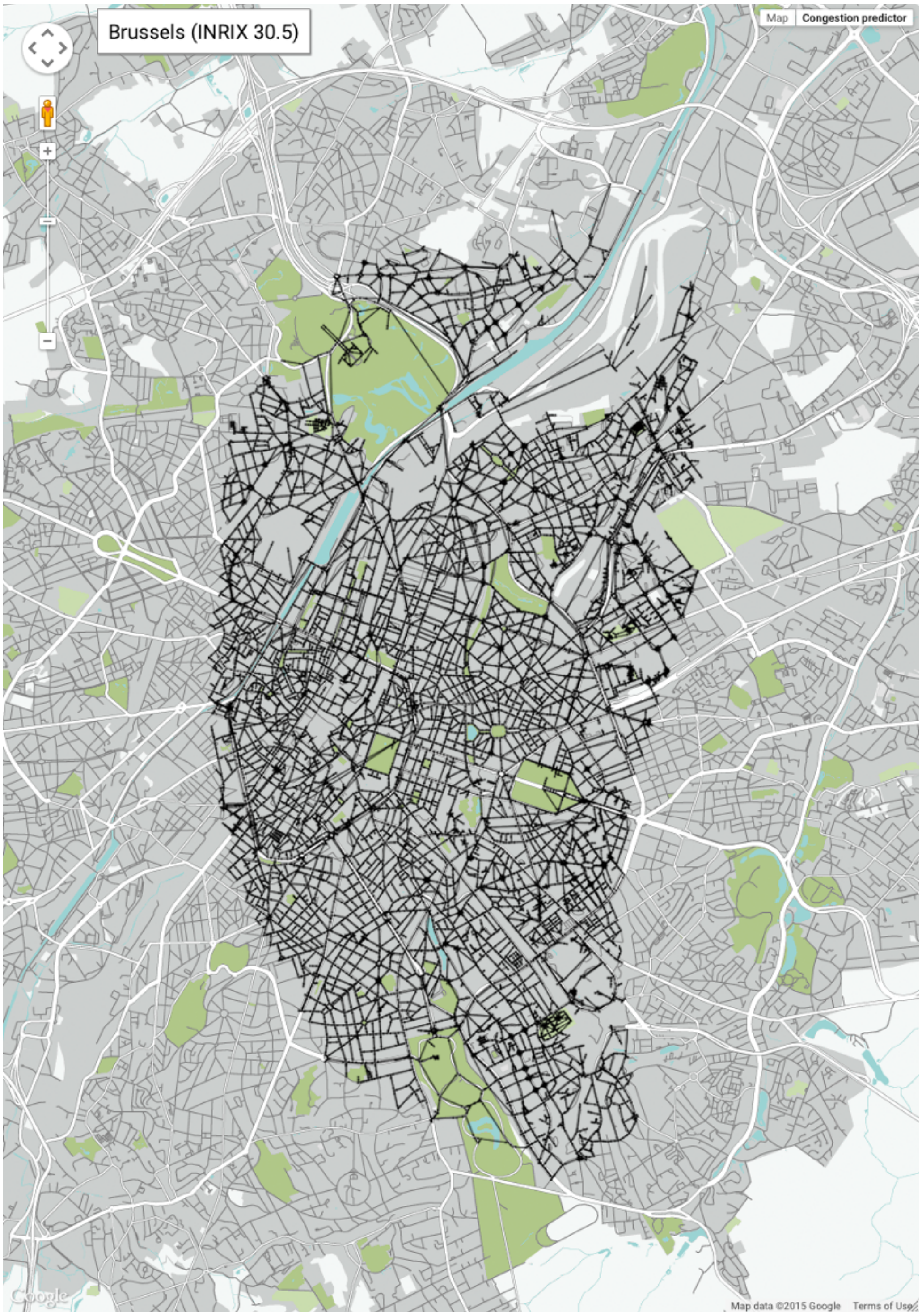

Supplementary Figure 7: Topology of the road network of Brussels. Data gathered from Open Street Map (http://www.openstreetmap.org).

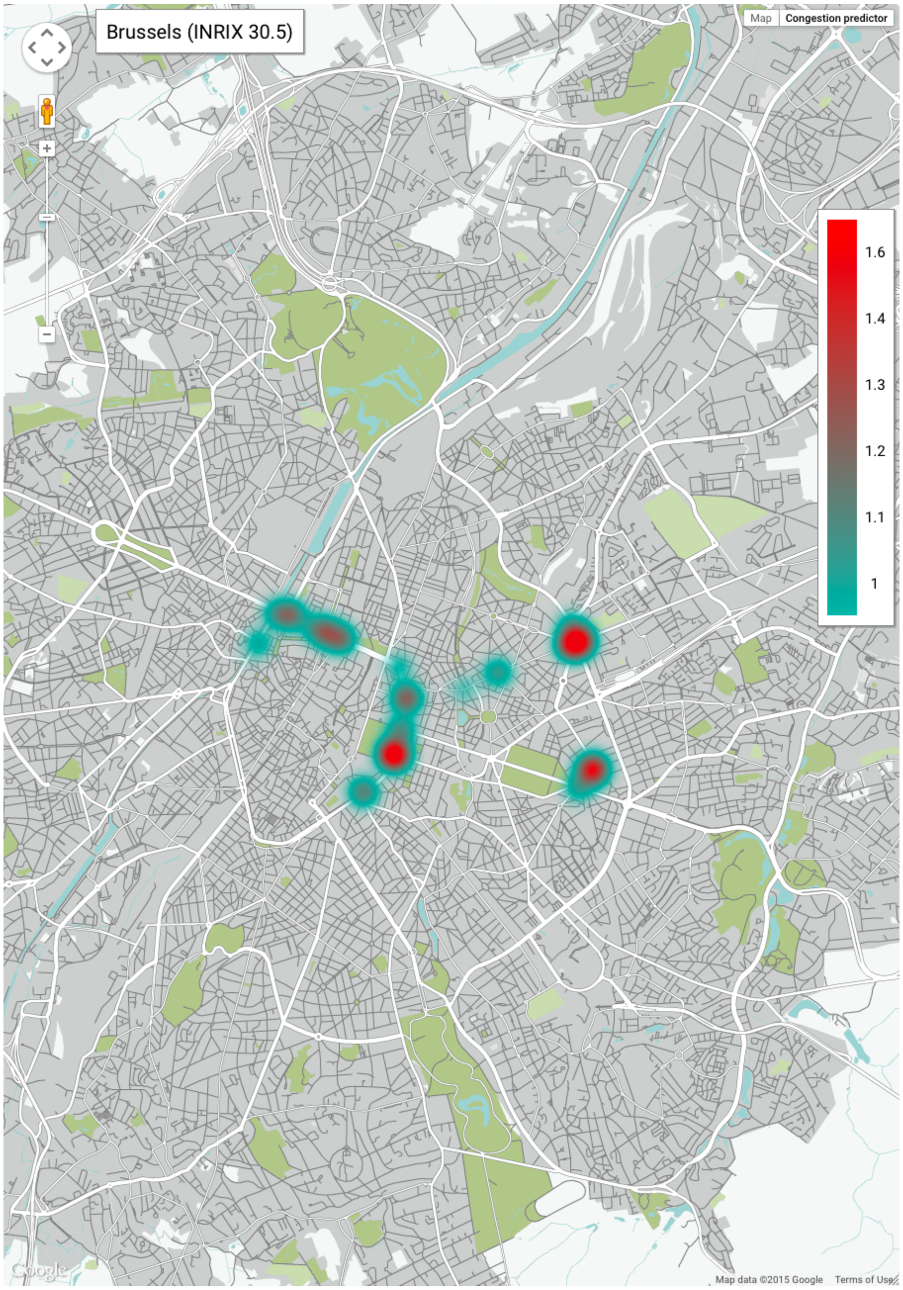

Supplementary Figure 8: Map of Brussels showing the expected ratio between incoming and outgoing vehicles of each congested junction, $(\sigma_i + g_i)/d_i$. Several congested junctions might appear merged in the heat map. Model parameters have been obtained assuming homogeneously distributed source and destination locations and the required road traffic to obtain an order parameter η [1, 2] equivalent to the city INRIX value.

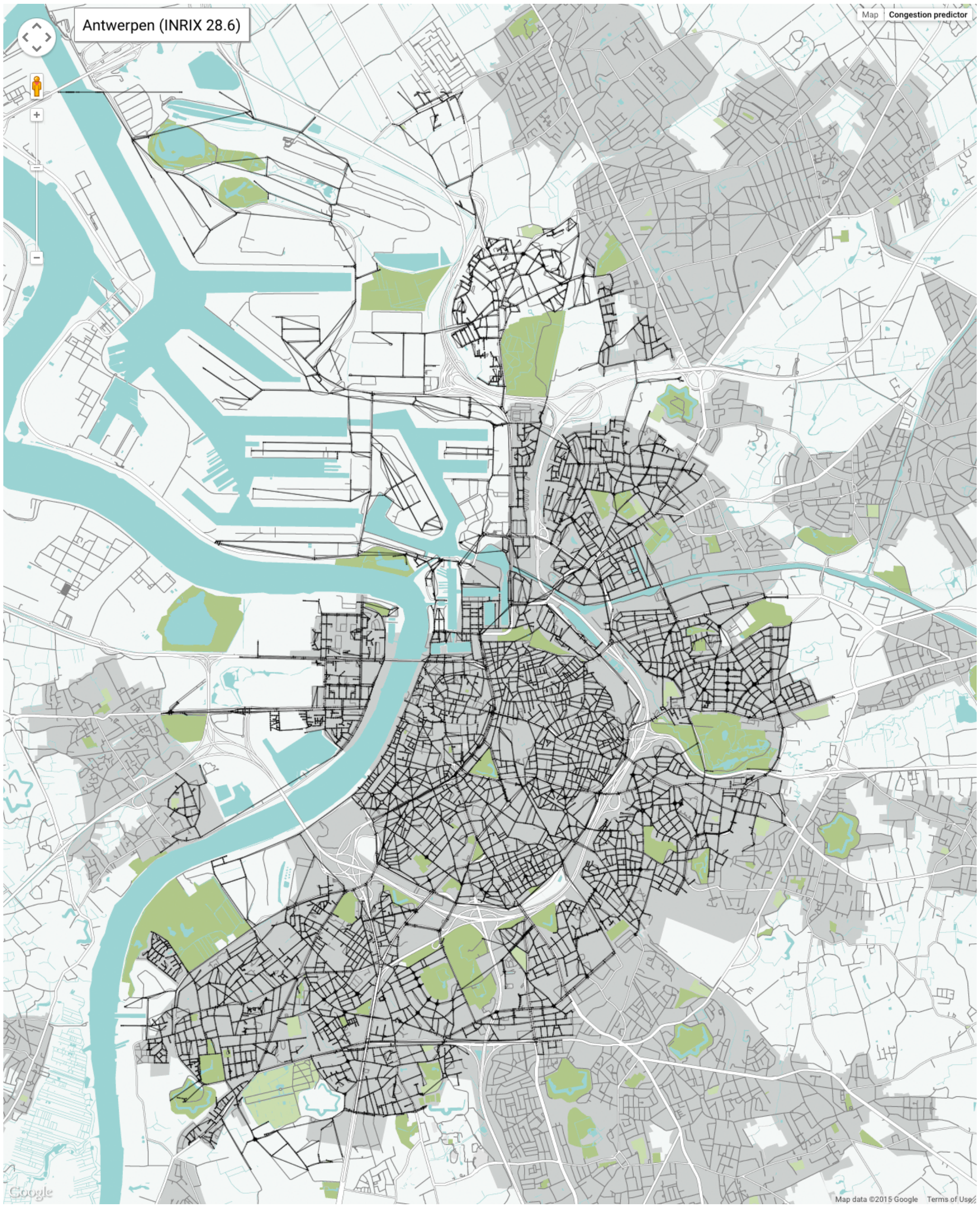

Supplementary Figure 9: Topology of the road network of Antwerpen. Data gathered from Open Street Map (http://www.openstreetmap.org).

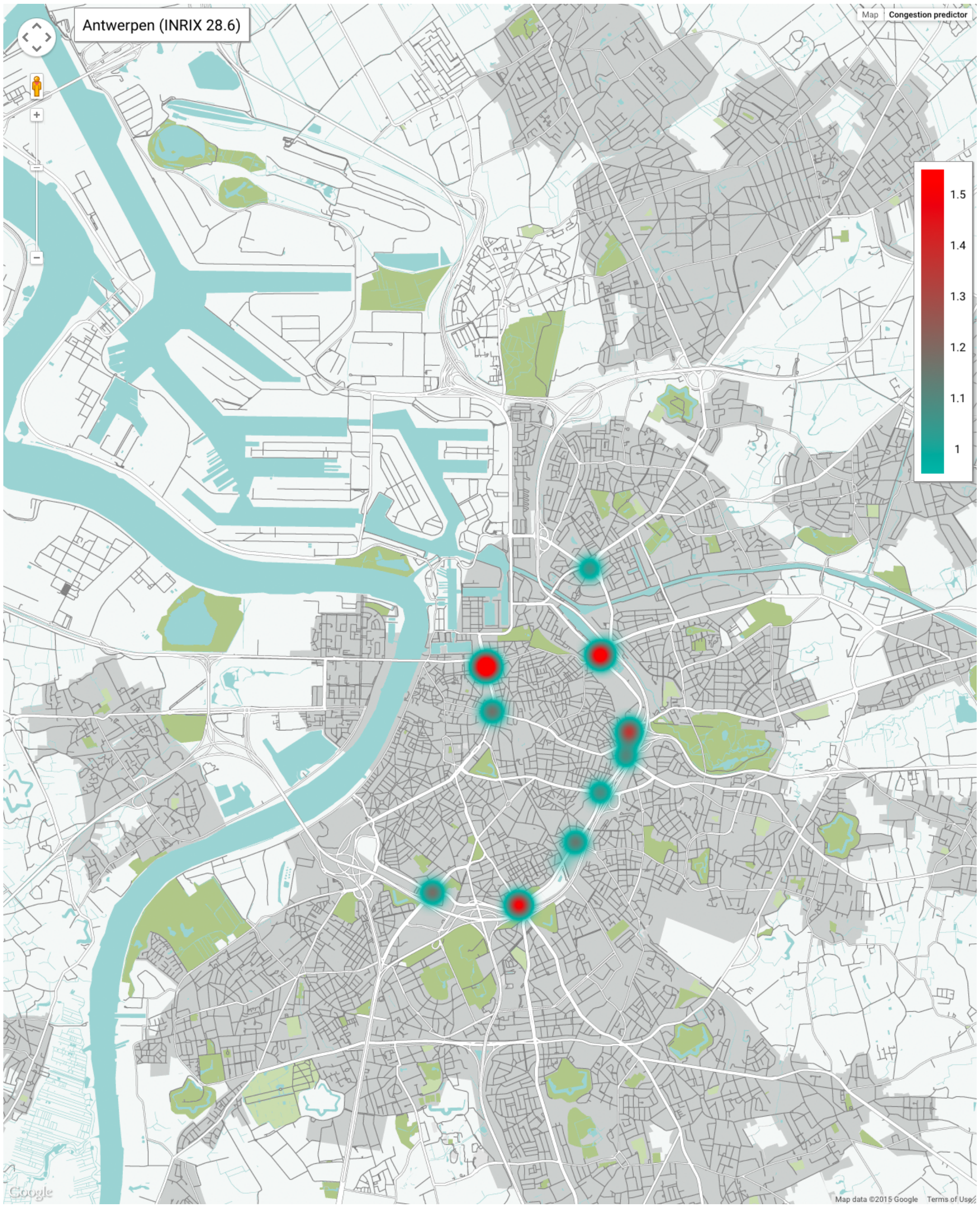

Supplementary Figure 10: Map of Antwerpen showing the expected ratio between incoming and outgoing vehicles of each congested junction, $(\sigma_i + g_i)/d_i$. Several congested junctions might appear merged in the heat map. Model parameters have been obtained assuming homogeneously distributed source and destination locations and the required road traffic to obtain an order parameter η [1, 2] equivalent to the city INRIX value.

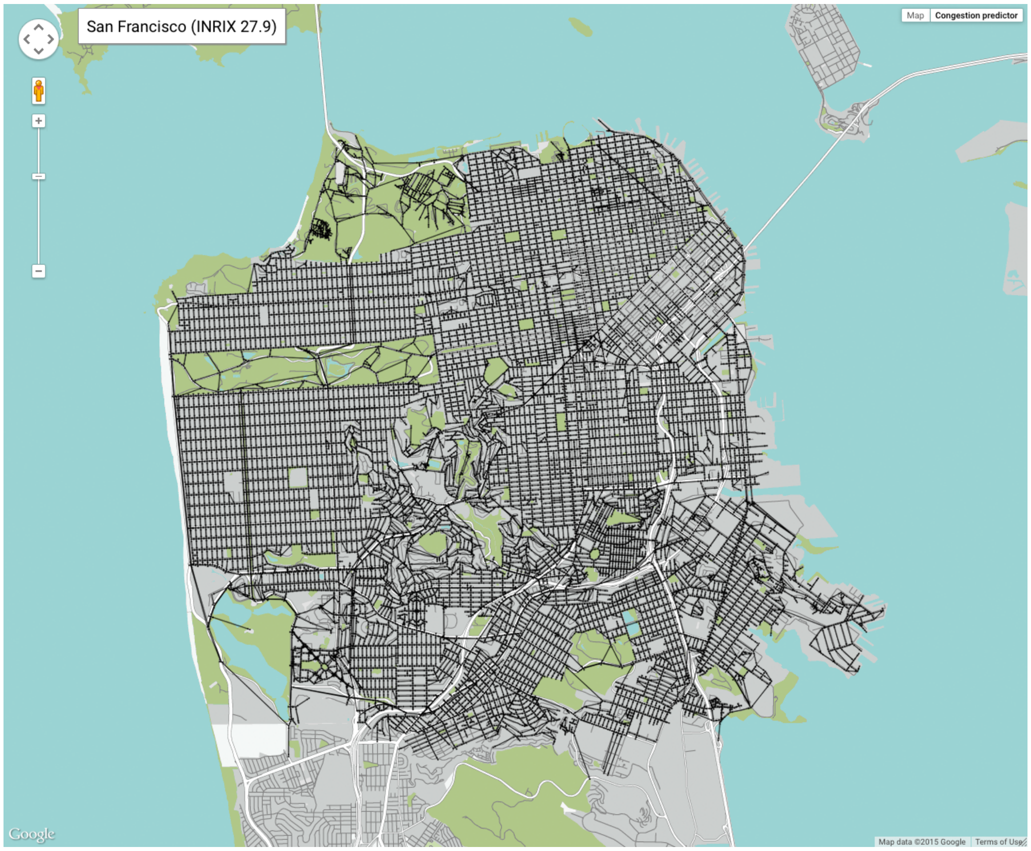

Supplementary Figure 11: Topology of the road network of San Francisco. Data gathered from Open Street Map (http://www.openstreetmap.org).

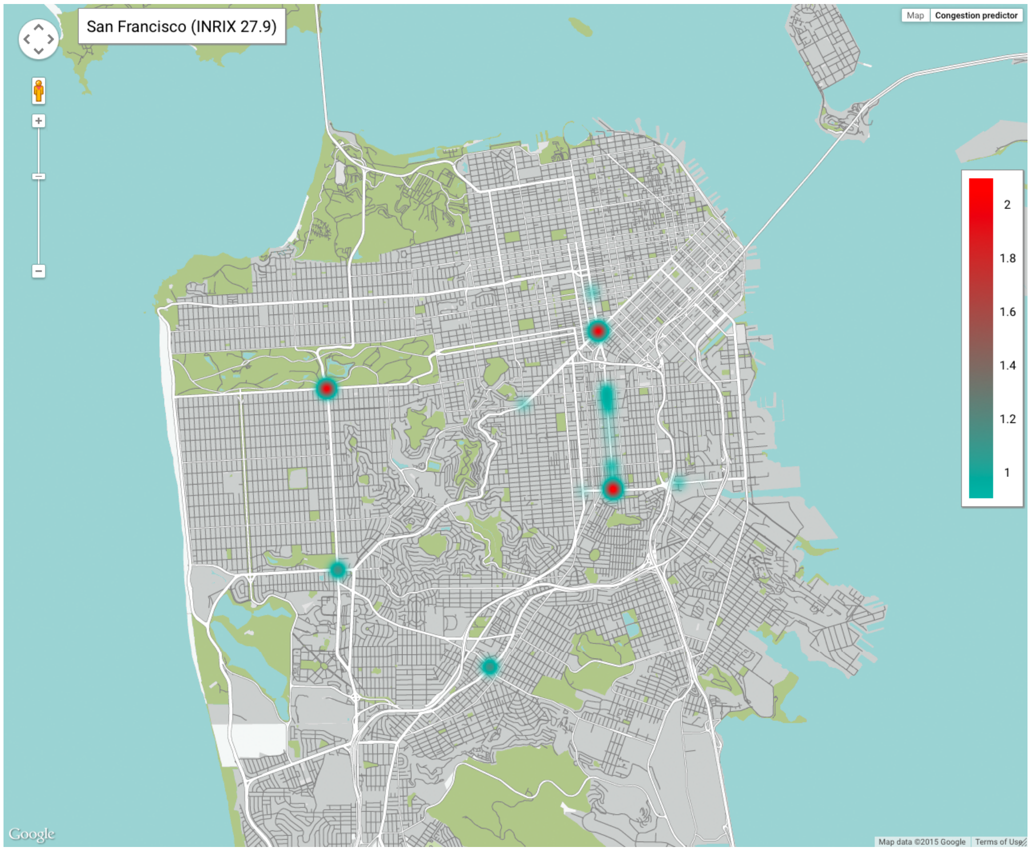

Supplementary Figure 12: Map of San Francisco showing the expected ratio between incoming and outgoing vehicles of each congested junction, $(\sigma_i + g_i)/d_i$. Several congested junctions might appear merged in the heat map. Model parameters have been obtained assuming homogeneously distributed source and destination locations and the required road traffic to obtain an order parameter η [1, 2] equivalent to the city INRIX value.

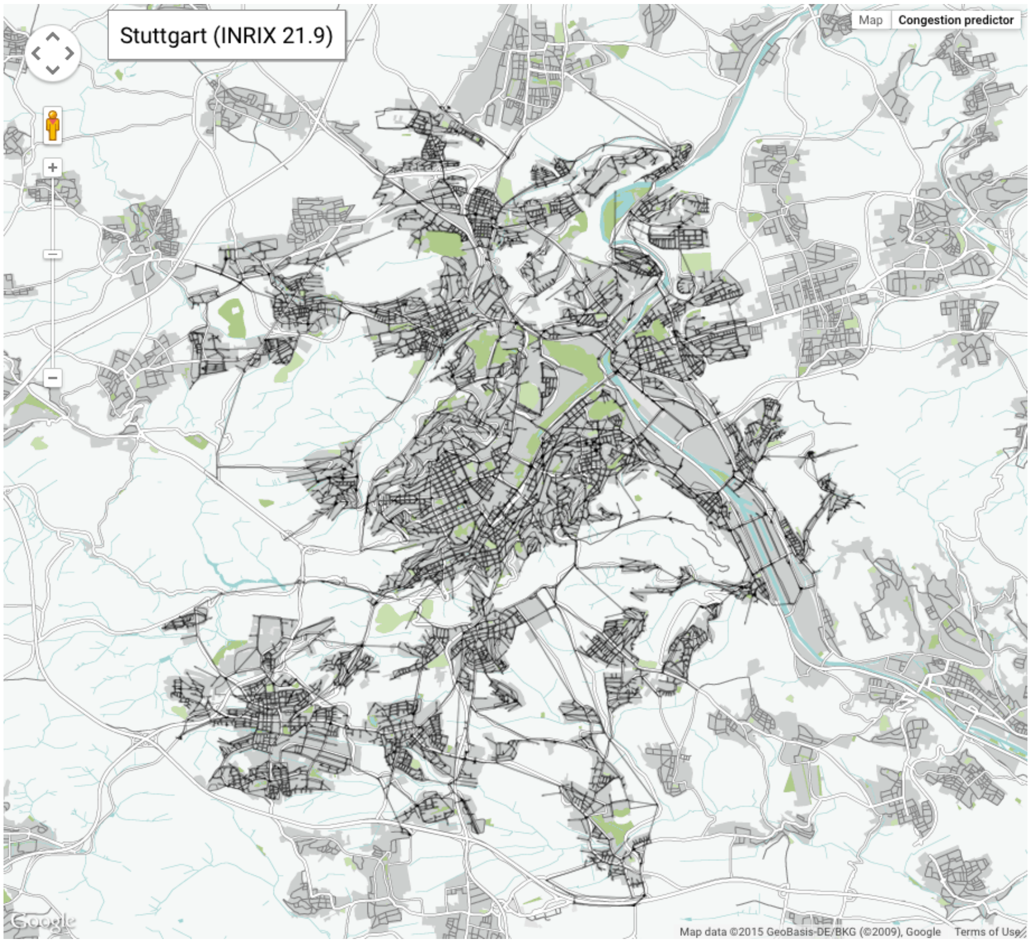

Supplementary Figure 13: Topology of the road network of Stuttgart. Data gathered from Open Street Map (http://www.openstreetmap.org).

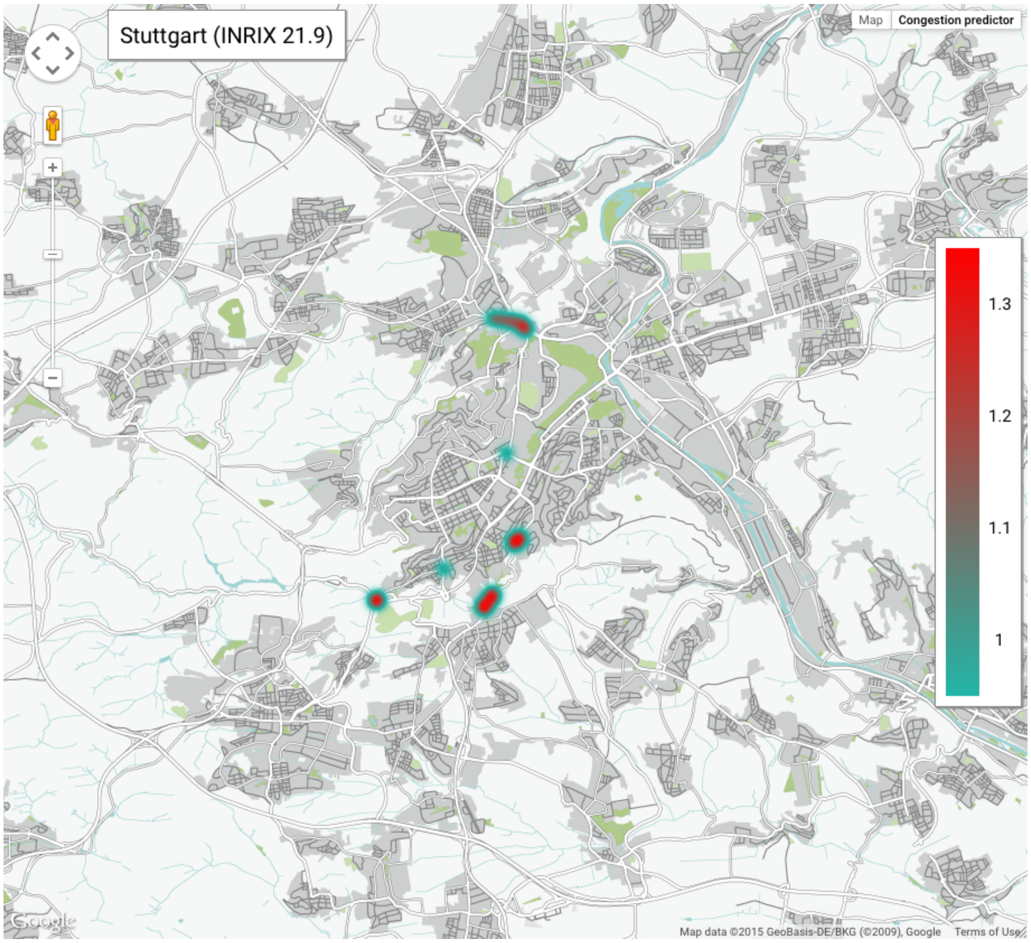

Supplementary Figure 14: Map of Stuttgart showing the expected ratio between incoming and outgoing vehicles of each congested junction, $(\sigma_i + g_i)/d_i$. Several congested junctions might appear merged in the heat map. Model parameters have been obtained assuming homogeneously distributed source and destination locations and the required road traffic to obtain an order parameter η [1, 2] equivalent to the city INRIX value.

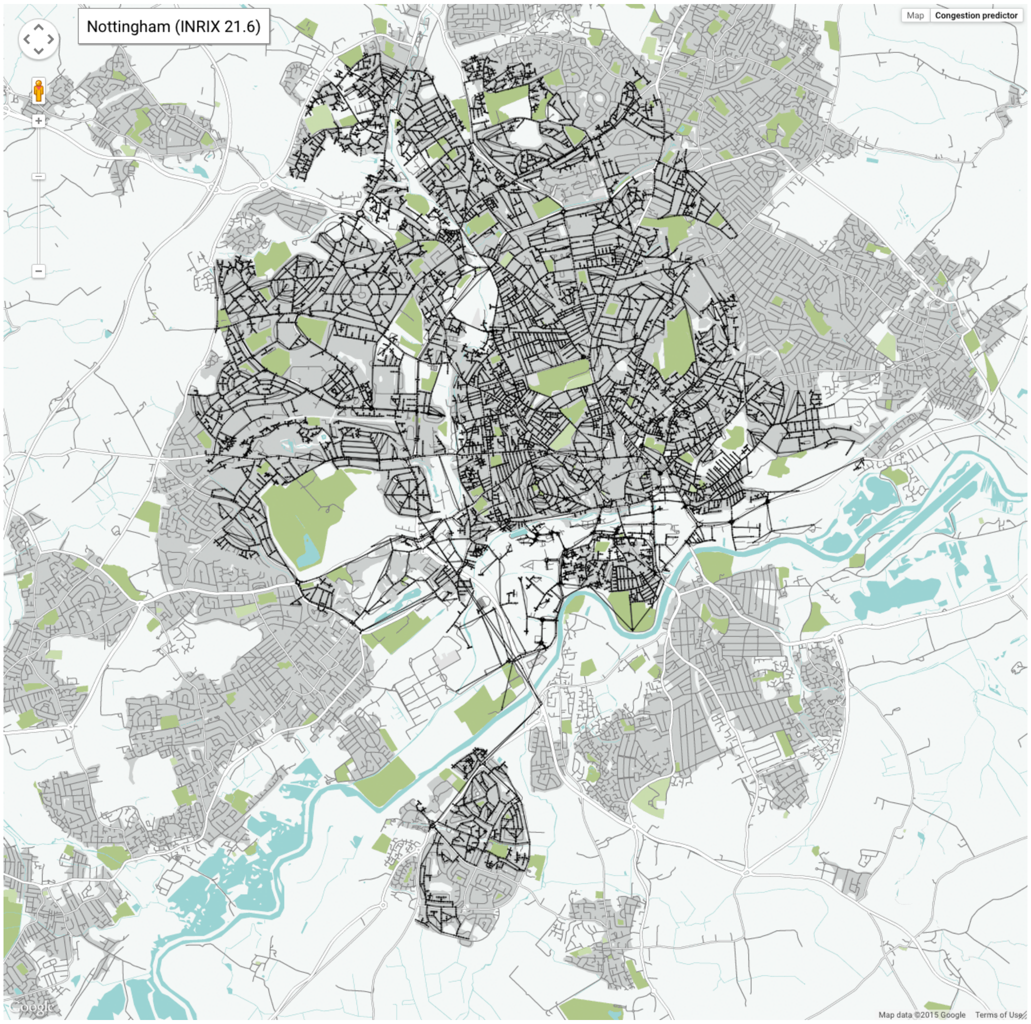

Supplementary Figure 15: Topology of the road network of Nottingham. Data gathered from Open Street Map (http://www.openstreetmap.org).

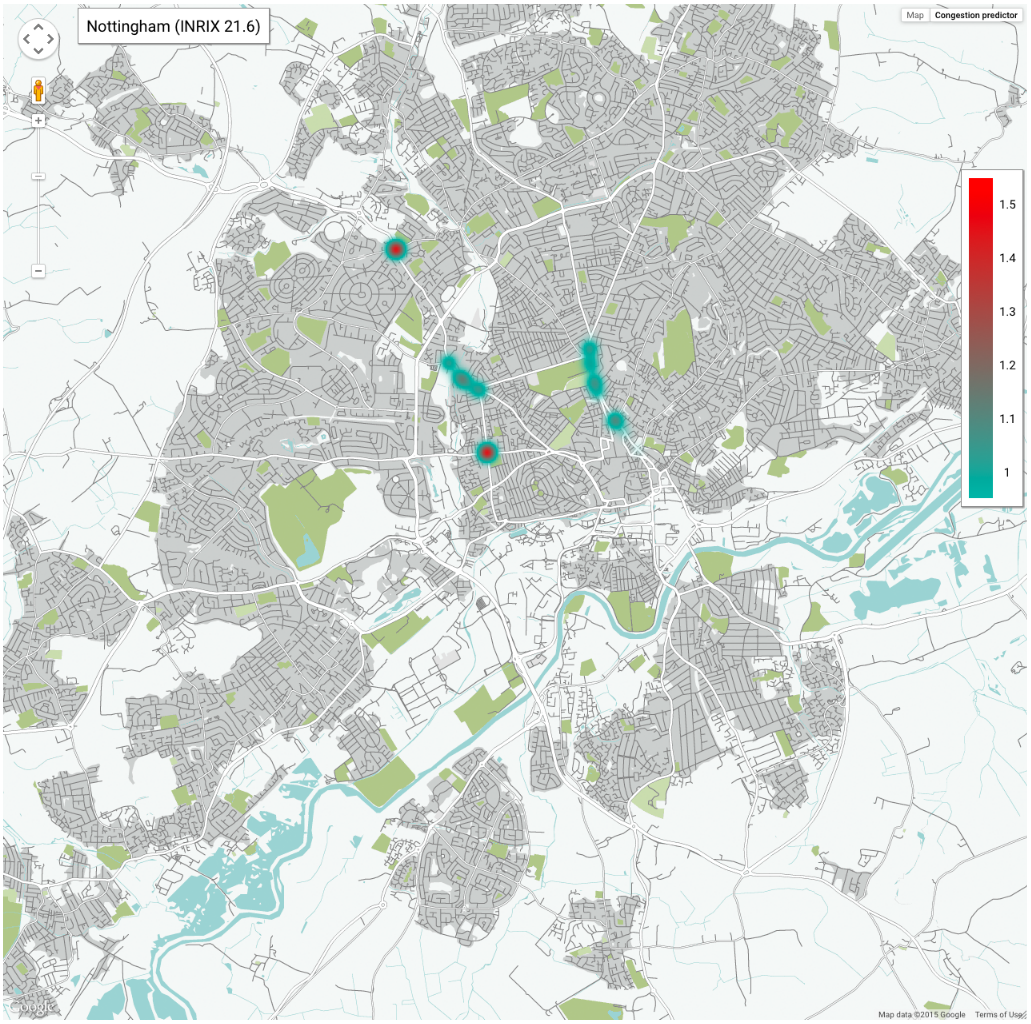

Supplementary Figure 16: Map of Nottingham showing the expected ratio between incoming and outgoing vehicles of each congested junction, $(\sigma_i + g_i)/d_i$. Several congested junctions might appear merged in the heat map. Model parameters have been obtained assuming homogeneously distributed source and destination locations and the required road traffic to obtain an order parameter η [1, 2] equivalent to the city INRIX value.

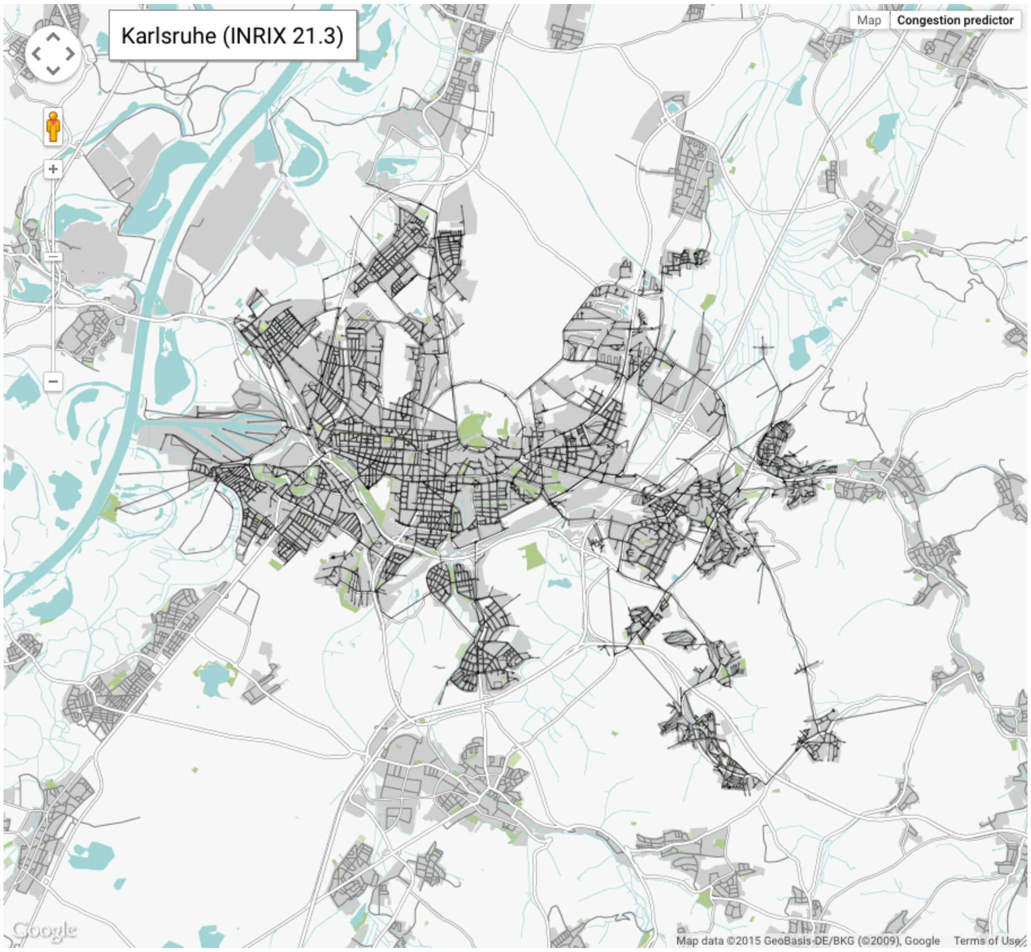

Supplementary Figure 17: Topology of the road network of Karlsruhe. Data gathered from Open Street Map (http://www.openstreetmap.org).

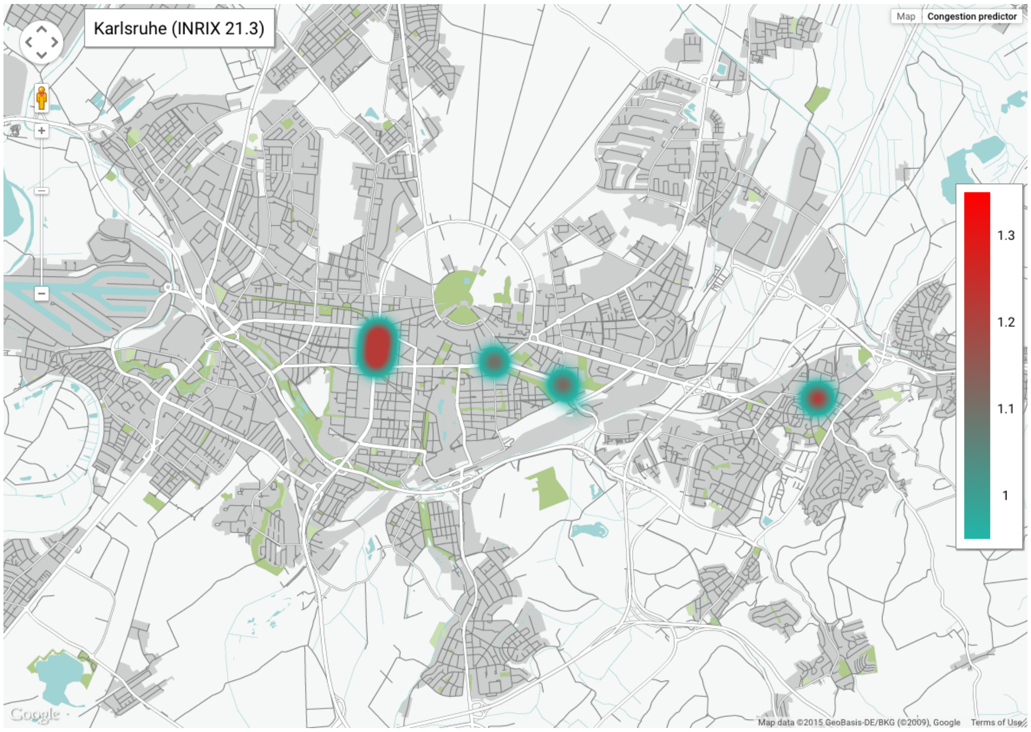

Supplementary Figure 18: Map of Karlsruhe showing the expected ratio between incoming and outgoing vehicles of each congested junction, $(\sigma_i + g_i)/d_i$. Several congested junctions might appear merged in the heat map. Model parameters have been obtained assuming homogeneously distributed source and destination locations and the required road traffic to obtain an order parameter η [1, 2] equivalent to the city INRIX value.

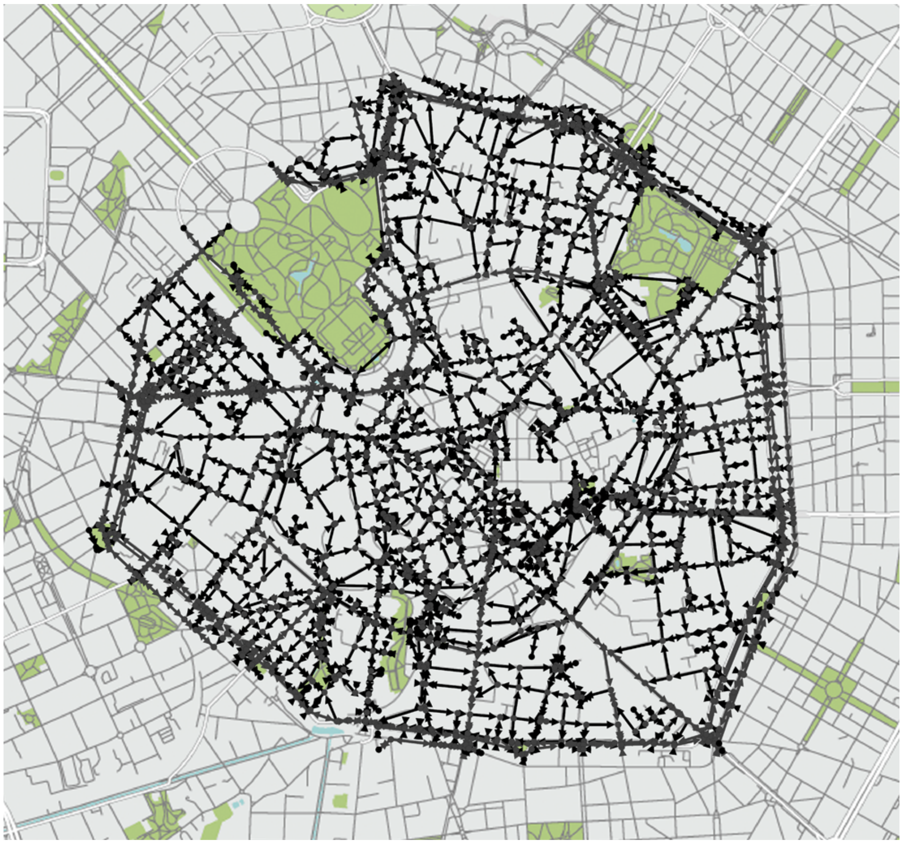

Supplementary Figure 19: Topology of the road network inside the Area-C of Milano. Data gathered from Open Street Map (http://www.openstreetmap.org).

Supplementary Note 1. Validation of the Microscopic Congestion Model

To validate MCM we run experiments on several synthetic networks and with two different routing strategies: local search strategy and shortest path strategy. In both routing strategies we assume, for simplicity, that all vehicles randomly choose the starting and ending junctions of their journey uniformly within all junctions of the network. Thus, each junction generates new vehicles with the same rate $g_i = \rho$. With the local search strategy vehicles do not have knowledge of the transportation network and traverse the network at random until they find their destination. For shortest path strategy, vehicles follow a randomly selected shortest path towards the destination. Without loss of generality we fix $\tau = 1$ and analyze the performance of MCM for different values of ρ.

For the local search strategy, the transition probabilities simplify to

$$P_{ji} = P_{ji}^{\mathrm{loc}} = P_{ji}^{\mathrm{ext}} = \frac{1}{k_j^{\mathrm{out}}} \,, \tag{1}$$

where k_j^{out} is the number of outgoing neighbours of node j. This means that, in this case, P_{ji} is independent of the congestion of the system.

Supplementary Figs. **20**, **21**, **22** and Fig. 2 of the main document show the performance of our model for different network topologies with the local search and shortest path routing strategies, respectively. As we see in the figures, the MCM achieves high accuracy in predicting the value of the order parameter η, probabilities p_i and P_{ij} (see *Materials and Methods* subsection *Microscopic Congestion Model*) as well as a perfect individual matching between the predicted and simulated values of the outgoing junction rates d_i.

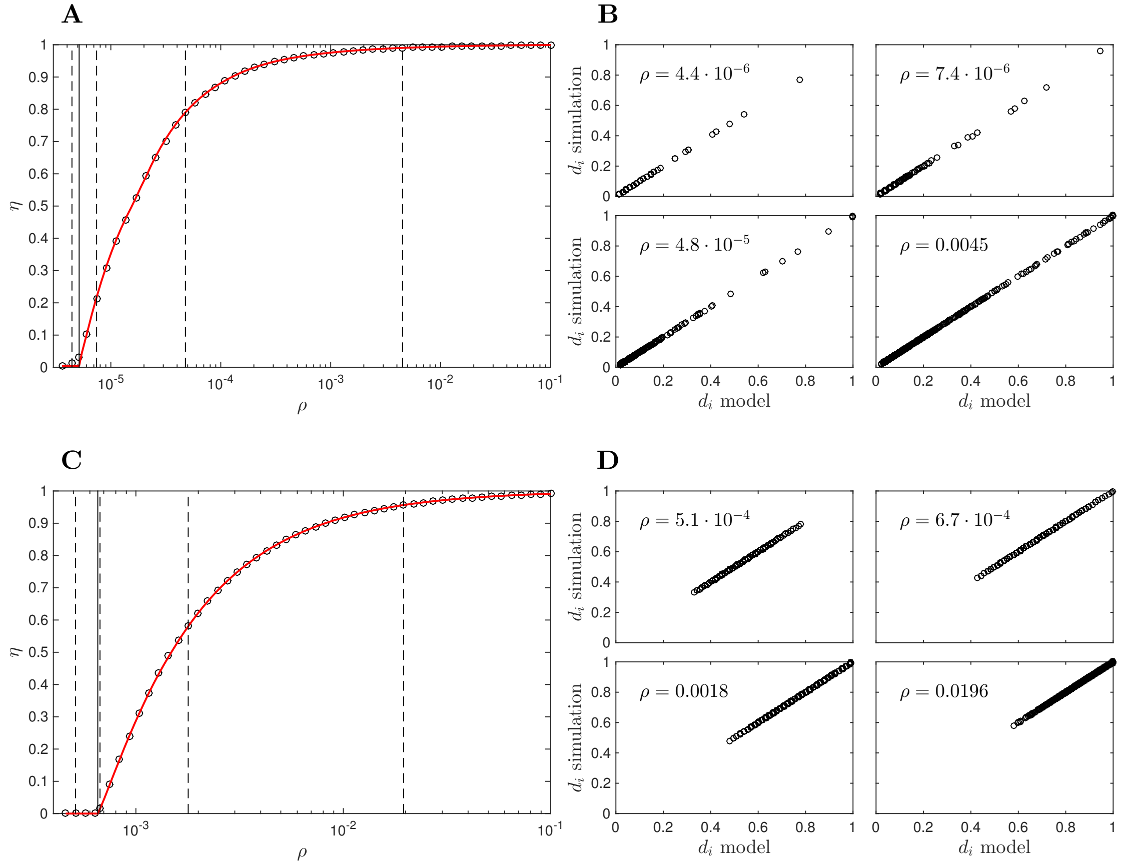

Supplementary Figure 20: Validation of the Microscopic Congestion Model with Barabási-Albert (**A,B**) and Erdős-Rényi (**C,D**) networks of 1000 nodes and local search routing strategy. In the construction procedure of the Barabási-Albert networks each new node is connected to 1 existing node in the network. The Erdős-Rényi networks have an average degree of 50. In **A** and **C**, accuracy in predicting the order parameter η. In **B** and **D**, correlation between predicted and simulated values of d_i. Vertical solid lines on plot **A** and **C** show the predicted critical generation rate ρ_c (see *Materials and Methods* subsection *Onset of congestion using the Microscopic Congestion Model*). Vertical dashed lines show the ρ values where d is evaluated on the left panels.

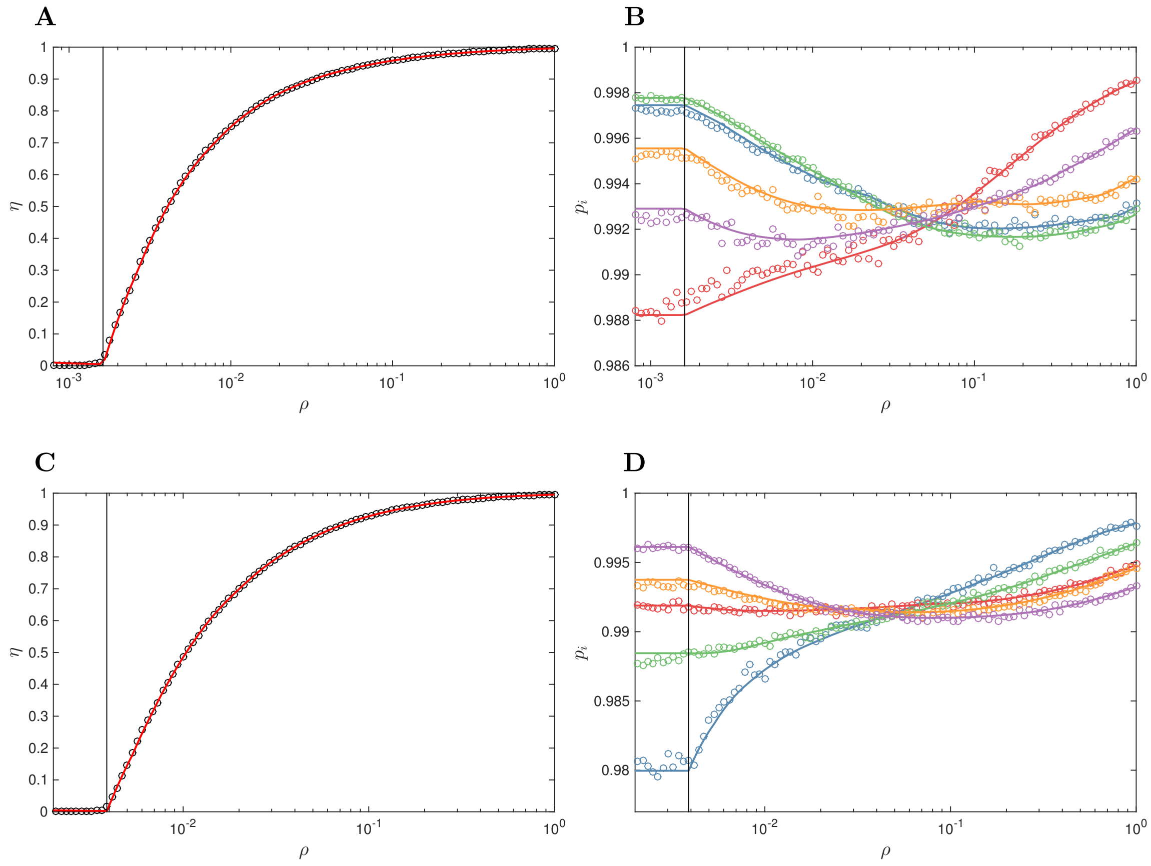

Supplementary Figure 21: Validation of the Microscopic Congestion Model with Barabási-Albert (**A,B**) and Erdős-Rényi (**C,D**) networks of 300 nodes and local search routing strategy. In the construction procedure of the Barabási-Albert networks each new node is connected to 1 existing node in the network. The Erdős-Rényi networks have an average degree of 15. In **A** and **C**, accuracy in predicting the order parameter η. In **B** and **D**, accuracy on predicting some of the probabilities p_i (see *Materials and Methods* subsection *Microscopic Congestion Model*). We have selected the probabilities that maximize the readability of the plot. Vertical solid lines on plot **A** and **C** show the predicted critical generation rate ρ_c (see *Materials and Methods* subsection *Onset of congestion using the Microscopic Congestion Model*).

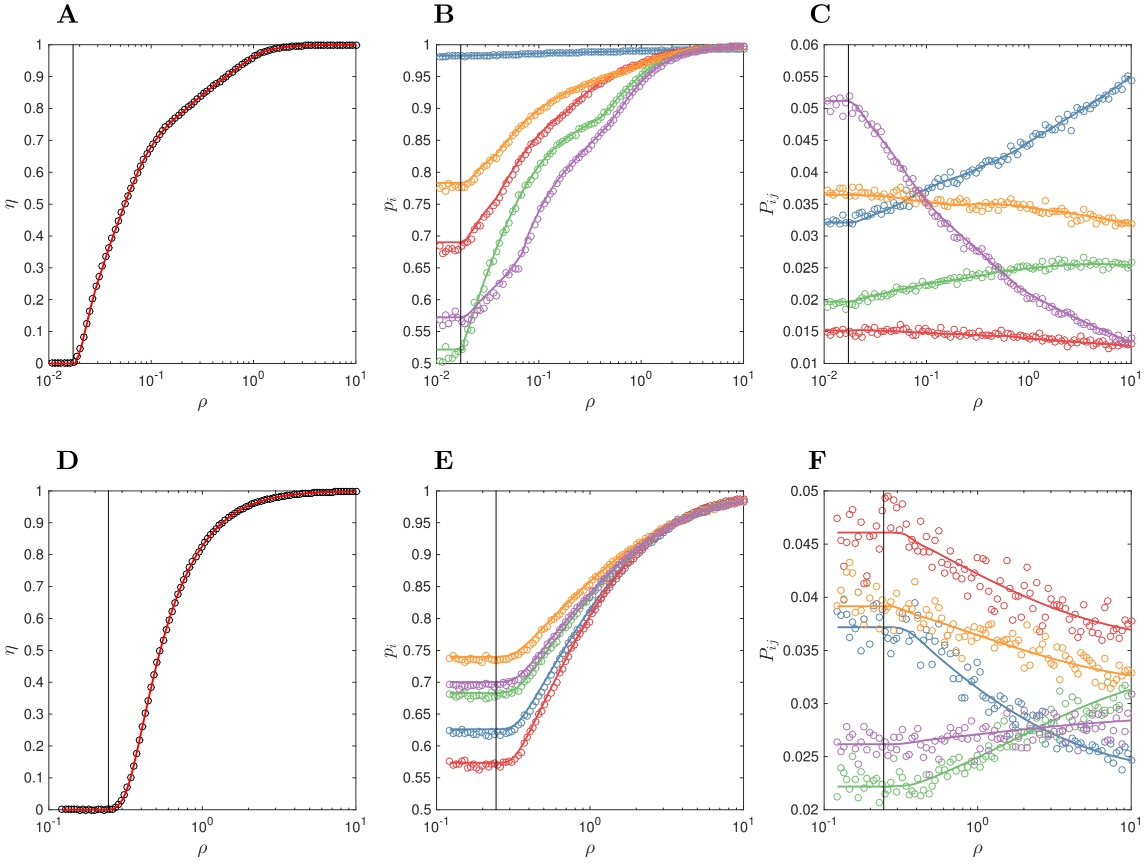

Supplementary Figure 22: Validation of the Microscopic Congestion Model with Barabási-Albert (**A, B, C**) and Erdős-Rényi (**D, E, F**) networks of 300 nodes and shortest path routing strategy. In the construction procedure of the Barabási-Albert networks each new node is connected to 1 existing node in the network. The Erdős-Rényi networks have an average degree of 15. In **A** and **D**, accuracy in predicting the order parameter η. In **B**, **C**, **D** and **F**, accuracy on predicting some of the probabilities p_i and P_{ij} (see *Materials and Methods* subsection *Microscopic Congestion Model*). We have selected the probabilities that maximize the readability of the plots. Vertical solid lines on plot **A** and **D** show the predicted critical generation rate ρ_c (see *Materials and Methods* subsection *Onset of congestion using the Microscopic Congestion Model*).

Supplementary References

[1] Arenas, A. *et al.* Optimal information transmission in organizations: search and congestion. *Review of Economic Design* **14**, 75–93 (2010).

[2] Guimerà, R., Díaz-Guilera, A., Vega-Redondo, F., Cabrales, A. & Arenas, A. Optimal network topologies for local search with congestion. *Physical review letters* **89**, 248701 (2002).
